# Shared genetic aetiology of puberty timing between sexes and with health-related outcomes

**DOI:** 10.1038/ncomms9842

**Published:** 2015-11-09

**Authors:** Felix R. Day, Brendan Bulik-Sullivan, David A. Hinds, Hilary K. Finucane, Joanne M. Murabito, Joyce Y. Tung, Ken K. Ong, John R.B. Perry

**Affiliations:** 1 MRC Epidemiology Unit, University of Cambridge School of Clinical Medicine, Box 285 Institute of Metabolic Science, Cambridge Biomedical Campus, Cambridge CB2 0QQ, UK; 2 Stanley Center for Psychiatric Research, Broad Institute of MIT and Harvard, Cambridge, Massachusetts 02142, USA; 3 Analytic and Translational Genetics Unit, Department of Medicine, Massachusetts General Hospital, Boston, Massachusetts 02114, USA; 4 Medical and Population Genetics, Broad Institute, Cambridge, Massachusetts 02142, USA; 5 23andMe Inc., 899 W. Evelyn Avenue, Mountain View, California 94041, USA; 6 Department of Epidemiology, Harvard School of Public Health, Boston, Massachusetts 02115, USA; 7 Department of Mathematics, Massachusetts Institute of Technology, Cambridge, Massachusetts 02139, USA; 8 NHLBI's and Boston University's Framingham Heart Study, Framingham, Massachusetts 01702-5827, USA; 9 Boston University School of Medicine, Department of Medicine, Section of General Internal Medicine, Boston, Massachusetts 02118, USA; 10 Department of Paediatrics, University of Cambridge, Cambridge CB2 0QQ, UK

## Abstract

Understanding of the genetic regulation of puberty timing has come largely from studies of rare disorders and population-based studies in women. Here, we report the largest genomic analysis for puberty timing in 55,871 men, based on recalled age at voice breaking. Analysis across all genomic variants reveals strong genetic correlation (0.74, *P*=2.7 × 10^−70^) between male and female puberty timing. However, some loci show sex-divergent effects, including directionally opposite effects between sexes at the *SIM1*/*MCHR2* locus (*P*_heterogeneity_=1.6 × 10^−12^). We find five novel loci for puberty timing (*P*<5 × 10^−8^), in addition to nine signals in men that were previously reported in women. Newly implicated genes include two retinoic acid-related receptors, *RORB* and *RXRA*, and two genes reportedly disrupted in rare disorders of puberty, *LEPR* and *KAL1.* Finally, we identify genetic correlations that indicate shared aetiologies in both sexes between puberty timing and body mass index, fasting insulin levels, lipid levels, type 2 diabetes and cardiovascular disease.

## Abstract (web summary)

Past studies on genetics of puberty relied on rare disorders or age of menarche in women. Here, Day *et al*. examine puberty timing in men by the age of voice breaking, and find some loci with sexually dimorphic effects and genetic architectures shared with other health conditions.

## Graphical abstract

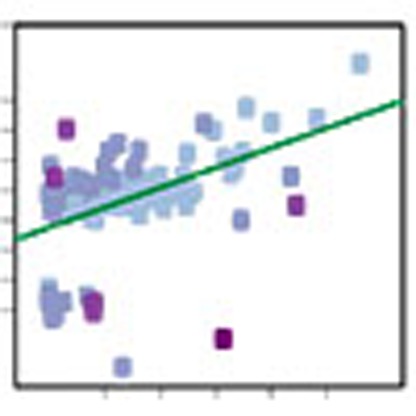

Voice breaking describes the drop in resonant frequency due to elongation of the larynx in response to androgen exposure^1^. It is a distinct developmental milestone that occurs during late puberty in males (typically between Tanner stages 3 to 4), and age at voice breaking therefore represents a non-invasive marker for the study of puberty timing in men^2^.

Age at menarche, the onset of the first menstrual bleed, is a similar marker of pubertal timing in females and has been more widely studied^3^. Age at menarche in women has been associated with a wide range of disease risks, and previous genome-wide association studies (GWASs) have reported over 100 common loci and five low-frequency coding variants, implicating several previously unsuspected mechanisms involved in the regulation of puberty timing, including post-transcriptional microRNA repression, gamma-aminobutyric acid-B (GABA-B) receptor signalling and nuclear hormone signalling^4^,^5^,^6^,^7^. Those menarche loci were reportedly enriched for variants also associated with puberty timing in boys, but those analyses were limited by the small number of boys with assessment of physical characteristics of puberty^5^,^6^.

Consequently, our understanding of the regulation of puberty timing in males is derived in large part from studies of rare disorders of puberty. To date, ∼20 genes have been implicated in abnormally delayed or absent puberty, including normosmic or anosmic hypogonadotrophic hypogonadism (Kallmann syndrome), while only three genes have been implicated in precocious puberty^8^,^9^. Here, we report the first large-scale GWAS of puberty timing in males, based on recalled age at voice breaking in men in the 23andMe study^10^. By combination with data on recalled age at menarche in women, we show that the genetic architecture of puberty timing has a substantial shared component between males and females, which also overlaps the genetic basis of several health-related traits and diseases.

## Results

### Genome-wide association signals for age at voice breaking

We identified 11 independent genome-wide significant (*P*<5 × 10^−8^) signals for age at voice breaking in men located at nine genomic loci (Table 1). Of these, nine signals (mapping to seven loci: in/near *LIN28B, MKL2, BSX, TMEM38B, NR4A2*, *IGSF1* and *ALMS1*) are correlated (*r*^2^>0.05) with reported loci for age at menarche in women^5^,^7^. The two novel signals for puberty timing are located in/near *LEPR* and *KAL1*, both of which are disrupted in rare disorders of puberty^8^. The strongest common signal (minor allele frequency (MAF)>5%) for voice breaking was at the *LIN28B* locus (rs9391253, *P*=8 × 10^−24^) where three independent signals were identified (Table 1), which is consistent with the allelic heterogeneity at this locus reported for age at menarche^5^. All signals are common variants, except for a rare (MAF=1%) intergenic variant near *ALMS1*, which is associated with 0.32 year per allele later age at voice breaking, and is highly correlated with a rare non-synonymous variant in *ALMS1* (rs45501594, T3542S, *r*^2^=0.83) that is reportedly associated with age at menarche in women^7^.

### Genetic overlap between puberty timing in men and women

**Figure 1:**
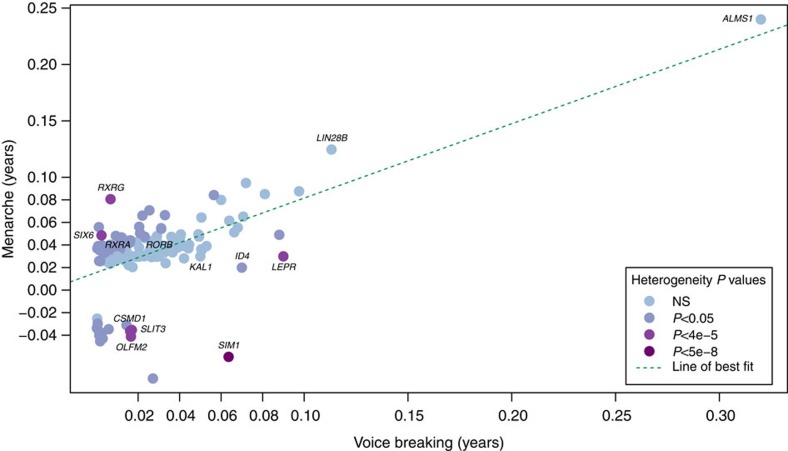
Scatterplot comparing effect sizes of the same genetic variants on age at menarche in women and age at voice breaking in men. Dots indicate newly identified voice breaking or previously reported age at menarche variants. NS, not significant.

To estimate the shared genetic aetiology between timing of puberty in men and women, we used LD Score Regression^11^ to calculate the genome-wide genetic correlation (*r*_g_) between age at voice breaking in men and age at menarche in women. The observed strong positive correlation (*r*_g_=0.74, *P*=2.7 × 10^−70^) indicates that many variants have similar influences on puberty timing in males and females. This sex concordance is illustrated by comparison of the effect sizes of all genome-wide significant loci for age at menarche/voice breaking (Fig. 1). Only six of the 123 reported age at menarche loci show significant sex-discordant effects on puberty timing (*P*_heterogeneity_<4.0 × 10^−4^); these were rs889122-*OLFM2*, rs466639-*RXRG*, rs2688325-*CSMD1*, rs1254337-*SIX6*, rs6555855-*SLIT3* and rs9321659-*SIM1*/*MCHR2.* Notably, at the *SIM1*/*MCHR2* locus the reported menarche-age raising allele is associated with younger age at voice breaking in men (*P*=7.9 × 10^−5^, *P*_heterogeneity_=1.6 × 10^−12^).

### Confirmation of novel puberty timing signals

Given the strong overall genetic correlation between puberty timing loci in men and women, and the paucity of male puberty timing data available in similarly sized studies, we sought confirmation of novel puberty timing loci identified in men using GWAS data on age at menarche in women (from a combination of publicly available HapMap2-imputed data from 182,416 women in the ReproGen consortium^5^ and 1,000-Genomes-imputed data from 76,831 additional women in the 23andMe study^10^,^12^).

Both of the novel genome-wide significant signals for age at voice breaking in men (in/near *LEPR* and *KAL1*) show directionally concordant associations with age at menarche in women (Table 1, *LEPR P*=1.85 × 10^−5^; *KAL1* 2.4 × 10^−4^). However, the *LEPR* signal (rs140410685 a 3 bp indel) shows a threefold larger effect on puberty timing in men than in women (*P*_heterogeneity_=3.7 × 10^−5^).

We next attempted to confirm sub-genome-wide significant signals for age at voice breaking (5 × 10^−8^<*P*<1 × 10^−6^). Of the five signals at this threshold, three were strongly correlated with reported signals for age at menarche^5^, showing directionally concordant associations with puberty timing in both sexes (rs10980922-*ZNF483*, rs2282752-*WDR6* and rs6681737-*NR5A2*). The other two signals (rs9350100-*RNF144B/ID4* and rs6560352-*RORB*) showed directionally concordant associations with age at menarche (*P*<0.01), but with significant heterogeneity between sexes (Table 1). Given this heterogeneity we meta-analysed the two estimates in a random-effects model. Variants at the novel *RNF144B-ID4* locus reached genome-wide significance for puberty timing in men and women combined. Although rs6560352 did not reach significance in this meta-analysis (*P*=2.1 × 10^−6^), an uncorrelated variant (rs4237264, *r*^2^∼0) at this *RORB* locus was previously reported as a possible signal for age at menarche (*P*=9 × 10^−6^)^5^. In a combined meta-analysis, we robustly confirmed rs4237264 as a novel signal for puberty timing in men and women (*P*=2 × 10^−11^).

*RORB* encodes retinoic acid receptor (RAR)-related orphan receptor beta; notably *RORA* and *RXRG* (encoding retinoid X receptor gamma) were previously implicated in age at menarche^5^. We therefore tested single nucleotide polymorphisms (SNPs) within 500 kb of the remaining six RAR, RAR-related and retinoid X receptor (RXR) encoding genes for associations with puberty timing in our pooled sample of men and women, identifying one additional novel signal ∼350 kb downstream of *RXRA* (rs416390, *P*=2 × 10^−8^) and a further suggestive signal ∼330 kb upstream of *RXRB* (rs241438, *P*=5 × 10^−6^). In aggregate, SNPs in or near a reported list of nuclear hormone receptor genes that contain these RAR-related and RXR genes are significantly enriched for associations with age at voice breaking in men (*P*=7 × 10^−3^), as reported for age at menarche in women^5^.

### Genetic correlation between puberty timing and other traits

To inform the likely aetiological relevance of puberty timing in men and women to other health-related outcomes, we used LD Score Regression to test their genetic correlations (*r*_g_) with 27 other traits or complex diseases. There is no significant heterogeneity observed in genetic correlations between men and women. In men and women combined, significant genetic correlations (conservatively adjusted for multiple testing: *P*<1.85 × 10^−3^ (=0.05/27)) are observed between puberty timing and nine other traits or disease outcomes (Table 2). The strongest genetic correlation is with body mass index (BMI; *r*_g_=−0.34, *P*=4.6 × 10^−104^); further inverse genetic correlations are observed with polycystic ovary syndrome, fasting insulin levels, type 2 diabetes, triglyceride levels, cardiovascular disease and bone mineral density; and positive genetic correlations are observed with high-density lipoprotein cholesterol levels and adult height.

## Discussion

We report a large genetic study of puberty timing in males and females. Our findings are the first to quantify the strongly shared genetic basis for puberty timing between sexes, and this is consistent with the largely sex-concordant effects of disruptive mutations in rare disorders of puberty^8^,^9^. Accordingly, our findings support the validity of recalled age at voice breaking in men as a marker of puberty timing in epidemiological studies, consistent with the informative prospective assessment of this phenotype^2^.

Independent signals at seven loci previously identified for age at menarche in women^5^ (including three independent signals at the *LIN28B* locus) passed the genome-wide statistical significance threshold for age at voice breaking in men (Table 1). These included the strongest reported common and low-frequency signals for age at menarche at *LIN28B* and *ALMS1*, respectively, and other signals with relatively large effects. This observation is consistent with the largely shared genetic architecture for puberty timing between sexes.

The overlapping genetic architecture for puberty timing in men and women provided the rationale for a pooled meta-analysis across the sexes. Notably, we identified two novel signals, near *RORB* and *RXRA*, which add to the reported signals near to other retinoic acid receptor-related genes, *RORA* and *RXRG*^5^, and a fifth signal (near *RXRB*) showed sub-genome-wide significant association with puberty timing. The retinoic acid receptor-related and retinoid X receptors function as transcription factors that dimerize and regulate nuclear receptors to influence cell differentiation, development, circadian rhythm and metabolism^13^. Their receptor partners include the canonical receptors for oestrogen and androgens among other hormones and metabolites^14^, and their conformational changes may alter receptor sensitivity^15^. Collectively these findings strengthen the evidence for an aetiological role of retinoic acid and retinoid receptors in the regulation of puberty timing, although their relative importance to male versus female puberty remains to be established. Further studies are also needed to identify functional links between these allelic signals and specific gene and protein functions.

Three additional novel signals reached genome-wide significance for puberty timing, represented by variants in/near *RNF144B-ID4*, *LEPR* and *KAL1.* All three were associated with puberty timing in both men and women, two of which had stronger effects in males (*LEPR* and *RNF144B-ID4*). The signal at 6p22.3 resides in a gene desert with the two nearest genes, *ID4* and *RNF144B*, located 760 kb and 607 kb away, respectively. Notably, it also lies 852 kb from *KDM1B*, a gene in the same family of histone demethlyases highlighted previously for age at menarche (variants in/near *KDM3B*, *KDM4A* and *KDM4C* represented genome-wide significant signals)^5^. Variants near *KAL1* on Xp22.31 had not been highlighted by previous GWAS of female puberty timing due to paucity of X-chromosome data in those studies. rs5978985 is correlated with a reported signal for circulating free testosterone concentrations in males (*r*^2^=0.35 with rs5934505)^16^ and *KAL1* encodes the extracellular matrix glycoprotein anosmin-1 implicated in the embryonic migration of gonadotrophin releasing hormone and olfactory neurons. Deleterious mutations in *KAL1* cause X-linked Kallmann syndrome, characterized by hypogonadotropic hypogonadism and anosmia^8^. rs140410685 near *LEPR*, which encodes the leptin receptor, is uncorrelated with a reported neighbouring signal for age at menarche (*r*^2^∼0 with rs10789181)^5^, and shows no reported association with adult BMI in reported GWAS meta-analyses (rs2186245 *r*^2^=1 proxy, *P*_BMI_=0.33, *N*=233,888)^17^.

In rare disorders of puberty, disruptive mutations usually have similar effects on puberty timing in both sexes^8^,^9^. Notable exceptions are rare mutations in the genes that encode the pituitary hormones, follicle-stimulating hormone and luteinizing hormone, which disrupt puberty in females but not in males^18^. In normal populations, rapid postnatal weight gain predicts earlier puberty timing in both sexes, but the influence of low birth weight is apparent only in females^2^. We found that only a small minority of signals showed sex-discordant effects, most notably rs9321659 at the *SIM1*-*MCHR2* locus. *SIM1* encodes a transcription factor regulator of hypothalamic paraventricular nucleus development and function. Rare deleterious mutations cause hyperphagia and severe early onset obesity affecting both males and females^19^; however, rs9321659 is reportedly not associated with adult BMI (*P*=0.56)^17^. Overall, these data suggest that combining male and female data genome wide is likely to yield novel shared puberty loci, but care should be taken to assess for potential heterogeneity in the effects.

Finally, our observed genetic correlations indicate the relevance of puberty timing to later life health outcomes in both men and women. Consistent with traditional epidemiological evidence^20^,^21^,^22^, earlier puberty timing was genetically related to higher risks of adverse health-related outcomes, including higher BMI, polycystic ovary syndrome, type 2 diabetes, lipid profiles and cardiovascular disease; it was favourable only for bone mineral density. LD score regression is a powerful tool to identify potential causal relationships between traits; however, limitations include the inability to establish causal directions and to adjust for potential-mediating factors. The relationship between puberty timing and obesity risk is complex, with plausible bi-directional mechanisms. Previous studies have reported that genome-wide significant signals for higher BMI, both in combination and individually, are associated with earlier puberty timing in females^5^,^23^,^24^. Conversely, in the opposite direction, earlier puberty timing associated with the *LIN28B* rs314276 C-allele leads to faster adolescent weight gain and higher post-pubertal BMI, without effects on pre-pubertal BMI^25^. Further studies are required to explore whether health outcomes related to earlier puberty timing are fully mediated by higher BMI.

In summary, this large-scale assessment of the genetic architecture of puberty timing in males quantifies the extent of shared aetiology between sexes, extends the evidence implicating retinoic acid-related receptors in the regulation of puberty timing, and supports the relevance of puberty timing in both sexes to the aetiologies of various health-related outcomes.

## Methods

### Genome-wide association study for age at voice breaking

Genome-wide SNP data were generated from one or more of three genotyping arrays in up to 55,871 men aged 18 or older of European ancestry from the 23andMe study^10^,^12^, who reported their recalled age at voice breaking, by online questionnaire in response to the question ‘How old were you when your voice began to crack/deepen?' Participants answered into one of the predefined age bins (under 9, 9–10 years old, 11–12 years old, 13–14 years old, 15–16 years old, 17–18 years old, 19 years old or older), scored from 0 to 6. Genetic effect estimates from these 2-year bins were re-scaled to 1-year effect estimates post analysis. We previously validated the accuracy of this approach by comparing re-scaled 2-year estimates for age at menarche (recorded in the same way) to those obtained from studies recording age at menarche by year. No significant heterogeneity was detected across these two approaches for known menarche loci^7^. 23andMe participants provided informed consent to take part in this research under a protocol approved by Ethical and Independent Review Services, an institutional review board accredited by the Association for the Accreditation of Human Research Protection Programs. Before imputation, we excluded SNPs with Hardy–Weinberg equilibrium *P*<10^−20^, call rate <95%, or with large allele frequency discrepancies compared with European 1,000 Genomes reference data. Frequency discrepancies were identified by computing a 2 × 2 table of allele counts for European 1,000 Genomes samples and 2,000 randomly sampled 23andMe participants with European ancestry, and identifying SNPs with a χ^2^ *P*<10^−15^. Genotype data were imputed against the March 2012 ‘v3' release of 1,000 Genomes reference haplotype panel. Genetic association results were obtained from linear regression models assuming additive allelic effects. These models included as covariates—age and the top five genetically determined principal components to account for population structure. The reported SNP association test *P* values were computed from likelihood ratio tests. Results were further adjusted for a lambda GC value of 1.069 to correct for any residual test statistic inflation due to population stratification. LD score regression analysis^26^ also confirmed that principal component correction had appropriately controlled for inflation due to population stratification (pre-GC calculated intercept∼1) before the more conservative GC correction.

Independent signals were identified using a combination of distance-based clumping and approximate conditional analysis. Firstly, regions were defined on the basis of physical proximity, with the most strongly associated SNP representing the association signal for that region. We then tested for the presence of multiple statistically independent signals in each region using approximate conditional analysis implemented in GCTA^27^. Independent signals indicated by SNP *P* values <1 × 10^−6^ were considered in follow-up analyses. A signal was considered to be the same as a previously reported menarche locus if it had a pairwise *r*^2^>0.05.

### Combined analyses with other puberty data sets

We followed up these selected SNPs in two additional sources of data: reported publicly available HapMap2 reference panel-imputed GWAS results for age at menarche from 182,416 women in the ReproGen consortium^5^; and independent 1,000 genomes reference panel-imputed GWAS data for age at menarche in 76,831 women in the 23andMe study^7^,^10^.

These additional samples were considered in three analytical designs. Firstly, all data in women (*n*=259,247) were combined to estimate effects of the individual selected SNPs on age at menarche, using fixed-effects inverse variance-weighted meta-analysis with all effect estimates reported on a per year scale. Secondly, for genetic correlation analyses with age at menarche, women in ReproGen consortium cohorts genotyped by the custom ‘iCOGs' array were excluded to obtain a consistent sample size across GWAS SNPs (leaving data for analysis on 209,820 women). Thirdly, all GWAS results for puberty timing in men and women (*n*=315,118) were combined using inverse variance fixed-effects meta-analysis. LD Score Regression^11^,^26^ showed that combining GWAS data from men and women did not introduce substantial test statistic inflation due to possible relatedness between strata (cross-trait intercept 0.016, s.e. 0.005). Heterogeneity between men and women for individual SNP associations was quantified by the I^2^ statistic generated by METAL software. SNPs that demonstrated heterogeneity were analysed in a random-effects model implemented by Han and Eskin^28^.

### Genetic correlations

Genetic correlations (*r*_g_) were calculated between age at voice breaking in men, age at menarche in women, and 27 other complex traits/diseases in publicly available data sets using LD Score Regression^11^,^26^. Data sets used can be downloaded from http://www.med.unc.edu/pgc/downloads. Sample numbers in the studies of each trait are shown in Table 2. The only non-publicly available data set used was a polycystic ovary syndrome GWAS performed on 5,184 self-reported cases and 82,759 controls from the 23andMe study^29^. A conservative Bonferroni corrected *P* value threshold of 0<1.85 × 10^−3^ (=0.05/27) was used to define significant associations.

## Additional information

**How to cite this article:** Day, F. R. *et al*. Shared genetic aetiology of puberty timing between sexes and with health-related outcomes. *Nat. Commun.* 6:8842 doi: 10.1038/ncomms9842 (2015).

## Figures and Tables

**Table 1:** Genetic variants associated with age at voice breaking in men and age at menarche in women.

				Age at VB, *N*=55,871		AAM, Max *N*=259,247			Puberty timing (men+women combined), Max *N*=315,118				
Location	SNP (r2)*	Alleles†	Gene	Effect (s.e.)	*P* value	Effect (s.e.)	*P* value	*N*	Effect (s.e.)‡	*P*_fixed_‡	*P*_random_§	*N*	Het P||
*Genome-wide significant signal for VB at a known AAM locus*													
6q16.3	rs9391253 (0.83)	T/A/0.32	*LIN28B*	0.12 (0.01)	8.2 × 10^−24^	0.12 (0.005)	1.5 × 10^−163^	256,338	0.12 (0.004)	5 × 10^−183^	—	312,259	0.35
16p13.12	rs246185 (1)	C/T/0.33	*MKL2*	0.09 (0.01)	5.7 × 10^−14^	0.05 (0.005)	4.0 × 10^−25^	254,604	0.05 (0.004)	2.1 × 10^−35^	—	310,475	2 × 10^−3^
11q24.1	rs7110373 (0.64)	T/C/0.51	*BSX*	0.08 (0.01)	1.4 × 10^−13^	0.05 (0.004)	2.3 × 10^−33^	256,434	0.05 (0.004)	3.0 × 10^−41^	—	312,305	0.20
9q31.2	rs9408817 (0.98)	G/A/0.67	*TMEM38B*	0.07 (0.01)	1.6 × 10^−10^	0.10 (0.005)	8.1 × 10^−98^	256,462	0.09 (0.004)	1.1 × 10^−105^	—	312,333	0.06
Xq26.2	rs5932886 (0.42)	T/C/0.82	*IGSF1*	0.06 (0.01)	1.9 × 10^−10^	0.08 (0.01)	8.3 × 10^−12^	76,831	0.07 (0.007)	1.5 × 10^−20^	—	132,702	0.36
2p13.1	rs35327298 (0.83)	C/T/0.01	*ALMS1*	0.32 (0.06)	9.4 × 10^−9^	0.24 (0.04)	3.4 × 10^−8^	76,831	0.27 (0.03)	3.2 × 10^−15^	—	132,702	0.25
2q24.1	rs142058842 (0.54)	G/C/0.17	*NR4A2*	0.08 (0.01)	2.2 × 10^−8^	0.05 (0.007)	1.6 × 10^−12^	239,327	0.05 (0.006)	5.7 × 10^−15^	—	295,198	0.90
													
*Novel genome-wide significant signal for VB*													
1p31.3	rs140410685¶	I/D/0.82	*LEPR*	0.09 (0.01)	8.7 × 10^−11^	0.03 (0.006)	1.8 × 10^−5^	209,761	0.04 (0.006)	—	1.4 × 10^−12^	265,632	3.7 × 10^−5^
Xp22.31	rs5978985	G/T/0.5	*KAL1*	0.05 (0.008)	2.1 × 10^−9^	0.03 (0.009)	2.4 × 10^−4^	76,831	0.04 (0.006)	4.6 × 10^−12^	—	132,702	0.22
													
*Secondary signals*													
6q16.3#	rs187150974	A/T/0.47	*LIN28B*	0.08 (0.01)	2.3 × 10^−9^	0.11 (0.01)	1.0 × 10^−20^	76,831	0.10 (0.009)	2.6 × 10^−30^	—	132,702	0.54
6q16.3#	rs9391261	T/A/0.22	*LIN28B*	0.10 (0.02)	5.6 × 10^−10^	0.12 (0.01)	4.5 × 10^−24^	76,831	0.11 (0.01)	1.8 × 10^−32^	—	132,702	0.19
9q21.13	rs6560352**	G/A/0.84	*RORB*	0.07 (0.01)	9.5 × 10^−7^	0.02 (0.007)	0.01	209,736	0.03 (0.006)	—	2.1 × 10^−6^	265,607	9 × 10^−4^
													
*Additional novel loci for puberty timing in men and women*													
6p22.3	rs9350100††	C/T/0.19	*RNF144B-ID4*	0.07 (0.01)	1.3 × 10^−7^	0.02 (0.006)	0.003	207,035	0.03 (0.006)	—	4.4 × 10^−8^	262,906	4 × 10^−4^
9q21.13	rs4237264	G/A/0.27	*RORB*	0.03 (0.01)	0.006	0.03 (0.005)	9.3 × 10^−10^	207,055	0.03 (0.005)	2.0 × 10^−11^	—	262,926	0.98
9q34.2	rs416390	G/C/0.29	*RXRA*	0.01 (0.01)	0.32	0.03 (0.005)	2 × 10^−8^	256,467	0.02 (0.004)	2.4 × 10^−8^	—	312,338	0.26

AAM, age at menarche; VB, voice breaking.

^*^Pairwise *r*^2^ with the lead SNP from Perry *et al*.^5^, if a known locus. The age at menarche lead SNPs (in the order as above) are: rs7759938, rs246185, rs1461503, rs10453225, rs762080, rs45501594 and rs17236969. These age at menarche lead SNPs were used for the combined puberty timing analyses because the voice breaking lead 1,000-Genomes imputed SNPs were often not available in the Hapmap2-imputed data from Perry *et al*. The exceptions were at the ALMS1 and IGSF1 loci, where the voice breaking lead SNPs were analysed for age at menarche only in 23andMe data.

^†^Defined as age at voice breaking increasing-allele/decreasing-allele/increasing-allele frequency.

^‡^Test statistics from fixed-effects inverse variance-weighted meta-analysis.

^§^*P* value from a random-effects meta-analysis was calculated where significant heterogeneity was detected at a novel locus.

^||^Test for heterogeneity in effect estimates between age at voice breaking in men and age at menarche in women from fixed-effects models.

^¶^HapMap2 proxy SNP rs2186245 (*r*^2^=1) was used for age at menarche and for puberty timing in men and women combined.

^#^Voice breaking effect estimates for secondary signals are from conditional models, however the menarche and combined estimates are from univariate models.

^**^HapMap2 proxy SNP rs2007888 (*r*^2^=0.98) was used for age at menarche and for puberty timing in men and women combined.

^††^HapMap2 proxy SNP rs2842385 (*r*^2^=0.99) was used for age at menarche and for puberty timing in men and women combined.

**Table 2:** Genetic correlations between puberty timing and 27 health-related outcomes.

	Age at menarche (*n*=209,820)*			Age at voice breaking (*n*=55,871)*			Age at menarche and voice breaking, combined (*N*=265,691)*		
Outcome†	*r*_g_	s.e.	*P* value	*r*_g_	s.e.	*P* value	*r*_g_	s.e.	*P* value‡
Body mass index (∼234k)	−0.35	0.02	1.4E−91	−0.28	0.04	1.9E−13	−0.34	0.02	**4.6E**−**104**
HDL cholesterol (∼100k)	0.17	0.03	3.4E−09	0.16	0.06	5.6E−03	0.17	0.03	**4.1E**−**11**
Polycystic ovary syndrome (∼88k)	−0.25	0.05	7.2E−07	−0.43	0.09	4.0E−06	−0.29	0.04	**6.4E**−**11**
Adult height (∼253k)	0.13	0.02	2.5E−10	0.06	0.03	9.0E−02	0.11	0.02	**1.8E**−**10**
Fasting insulin (∼52k)	−0.23	0.05	3.6E−06	−0.26	0.09	2.5E−03	−0.24	0.04	**3.4E**−**08**
Triglycerides (∼97k)	−0.12	0.03	5.1E−06	−0.07	0.06	2.0E−01	−0.11	0.02	**2.7E**−**06**
Cardiovascular disease (87k)	−0.12	0.04	5.0E−03	−0.18	0.07	9.3E−03	−0.13	0.04	**1.9E**−**04**
Type 2 diabetes (69k)	−0.13	0.04	5.2E−04	−0.03	0.07	7.1E−01	−0.11	0.03	**1.1E**−**03**
Lumbar spine BMD (33k)	−0.08	0.03	1.4E−02	−0.12	0.06	3.5E−02	−0.09	0.03	**1.5E**−**03**
Femoral neck BMD (33k)	−0.08	0.03	1.3E−02	−0.10	0.05	6.8E−02	−0.08	0.03	2.2E−03
HbA1c (46k)	−0.11	0.05	2.1E−02	−0.16	0.09	7.8E−02	−0.12	0.04	4.1E−03
Fasting glucose (58k)	−0.06	0.04	8.7E−02	−0.11	0.07	9.3E−02	−0.07	0.03	2.0E−02
Bipolar disorder (17k)	0.07	0.04	9.2E−02	0.12	0.07	9.1E−02	0.08	0.04	2.3E−02
Autism (10k)	−0.10	0.04	2.3E−02	−0.04	0.08	6.5E−01	−0.09	0.04	2.8E−02
Schizophrenia (70k)	0.03	0.03	3.1E−01	0.11	0.05	1.6E−02	0.05	0.02	3.9E−02
Birth weight (27k)	−0.07	0.05	1.5E−01	−0.04	0.09	6.7E−01	−0.06	0.04	1.4E−01
Rheumatoid arthritis (26k)	0.04	0.04	3.8E−01	0.11	0.09	1.9E−01	0.05	0.04	1.7E−01
Alzheimer's disease (54k)	0.04	0.05	4.2E−01	0.10	0.10	3.3E−01	0.05	0.05	2.5E−01
Major depressive disorder (19k)	−0.04	0.06	4.7E−01	−0.11	0.11	3.3E−01	−0.05	0.05	2.8E−01
Crohn's disease (21k)	0.04	0.03	1.8E−01	−0.02	0.06	7.5E−01	0.03	0.03	3.0E−01
Diastolic blood pressure (69k)	−0.03	0.03	3.8E−01	−0.03	0.08	6.6E−01	−0.03	0.03	3.3E−01
ADHD (5k)	−0.06	0.08	4.2E−01	−0.03	0.14	8.5E−01	−0.05	0.07	4.3E−01
Systolic blood pressure (69k)	−0.03	0.03	4.5E−01	0.01	0.07	8.9E−01	−0.02	0.03	5.3E−01
Ever smoker (74k)	0.01	0.04	7.3E−01	0.04	0.07	5.9E−01	0.02	0.03	5.7E−01
LDL cholesterol (95k)	0.01	0.03	6.4E−01	0.00	0.06	9.9E−01	0.01	0.03	6.7E−01
Anorexia nervosa (18k)	0.03	0.04	4.3E−01	−0.05	0.07	5.0E−01	0.01	0.03	6.8E−01
Ulcerative colitis (27k)	0.04	0.05	4.1E−01	−0.04	0.07	5.7E−01	0.01	0.04	7.4E−01

ADHD, attention-deficit hyperactivity disorder; BMD, bone mineral density; HDL, high-density lipoprotein; LDL, low-density lipoprotein.

^*^Sample size in each GWAS for puberty timing.

^†^Sample size in each GWAS for health-related outcomes is shown in parentheses after each outcome name.

^‡^*P* values below the multiple test-corrected threshold<1.85 × 10^−3^ (=0.05/27) are highlighted in bold.
